# The Impact of Type 2 Diabetes on Bone Fracture Healing

**DOI:** 10.3389/fendo.2018.00006

**Published:** 2018-01-24

**Authors:** Carlos Marin, Frank P. Luyten, Bart Van der Schueren, Greet Kerckhofs, Katleen Vandamme

**Affiliations:** ^1^Skeletal Biology and Engineering Research Center, Department of Development and Regeneration, KU Leuven, Leuven, Belgium; ^2^Prometheus—Division of Skeletal Tissue Engineering Leuven, KU Leuven, Leuven, Belgium; ^3^Biomaterials—BIOMAT, Department of Oral Health Sciences, KU Leuven, Leuven, Belgium; ^4^Clinical and Experimental Endocrinology, Department of Clinical and Experimental Medicine, KU Leuven, Leuven, Belgium

**Keywords:** fracture healing, type 2 diabetes mellitus, bone regeneration, fracture risk, hyperglycemia, bone turnover

## Abstract

Type 2 diabetes mellitus (T2DM) is a chronic metabolic disease known by the presence of elevated blood glucose levels. Nowadays, it is perceived as a worldwide epidemic, with a very high socioeconomic impact on public health. Many are the complications caused by this chronic disorder, including a negative impact on the cardiovascular system, kidneys, eyes, muscle, blood vessels, and nervous system. Recently, there has been increasing evidence suggesting that T2DM also adversely affects the skeletal system, causing detrimental bone effects such as bone quality deterioration, loss of bone strength, increased fracture risk, and impaired bone healing. Nevertheless, the precise mechanisms by which T2DM causes detrimental effects on bone tissue are still elusive and remain poorly studied. The aim of this review was to synthesize current knowledge on the different factors influencing the impairment of bone fracture healing under T2DM conditions. Here, we discuss new approaches used in recent studies to unveil the mechanisms and fill the existing gaps in the scientific understanding of the relationship between T2DM, bone tissue, and bone fracture healing.

## Introduction

Type 2 diabetes mellitus (T2DM) is a very common comorbidity of obesity. It is characterized by hyperglycemia, resulting from insulin resistance and islet β-cell dysfunction (1). According to the World Health Organization (WHO), more than 422 million people are currently suffering from diabetes mellitus (DM), with T2DM accounting for 90% of these cases. WHO predicts DM to become the seventh leading cause of death in the world by 2030. Among its many complications, T2DM is known to cause a negative effect on the skeletal system (2–4). Currently, there are evidences suggesting that the process of bone healing after trauma (fracture) is compromised under T2DM conditions (5–7). Impaired vascularity and T2DM-enhanced inflammation impede the proper distribution of oxygen, nutrients, and osteoprogenitor cells to the repair site (8, 9). Cellular and molecular characteristics of the bone tissue are also altered under T2DM conditions in bone healing. For instance, it is suggested that not only the functionality of the osteoblasts (osteoprogenitors) may be compromised in the diabetic microenvironment but also that these cells are switching their differentiation fate toward the adipogenic lineage, increasing the amount of fat tissue in the fracture callus and thus hampering the fracture healing process (5, 10). Bone turnover has also been found to be altered in the presence of T2DM, having a negative impact in bone formation and/or bone resorption (11). Furthermore, the generation of advanced glycation end products (AGEs) due to the presence of hyperglycemia is capable of altering the bone matrix and reducing the bone quality (12–14). Despite these reported evidences, the exact mechanisms of the pathology that T2DM causes on bone fracture healing remains poorly understood. In this review, we revised the different T2DM-related factors that have been suggested to affect the bone fracture healing process and discussed recent findings to fill the current gaps in the scientific understanding of the impact of T2DM in bone repair.

## Methodology

### Focused Question

The following question was posed to define the content of this review article: what are the possible causes of impaired bone fracture healing in T2DM?

### Search Protocol

Articles related to the topic of this review and potentially contributing to answering the aforementioned proposed question were searched for, using the PubMed database of the US National Library of Medicine and National Institutes of Health. The key terms and their combinations input in the database for the search of potential articles are displayed in Table 1. The timeline for the selection of potentially relevant papers was set between January 2007 and October 2017.

**Table 1:** Key terms used for the literature search performed in the PubMed database.

Key terms used in the search protocol	Number of articles retrieved
Diabetes AND Fracture Healing	22
Type 2 Diabetes AND Fracture Healing	8
Diabetes AND Bone Regeneration	3
Diabetes AND Bone Healing	3
Diabetes AND Fracture Repair	4
Hyperglycemia AND Fracture Healing	4
Hyperglycemia AND Bone Regeneration	1
Diabetic Mice AND Fracture Healing	1
Diabetes AND Bone Formation	3
Hyperglycemia AND Bone Formation	2
Diabetes AND Non-union	2
Diabetes AND Callus Formation	6

### Eligibility Criteria

The criterion, on which the selection of the articles to be included in the review was based, was the study of the fracture healing process under T2DM conditions. Different categories of studies were considered, including animal studies, clinical studies, and review papers. Some articles outlining the principal mechanisms of bone healing in normal conditions were also included. Articles solely focusing on type 1 diabetes mellitus (T1DM) were excluded from the final selection.

### Search Process

**Figure 1:**
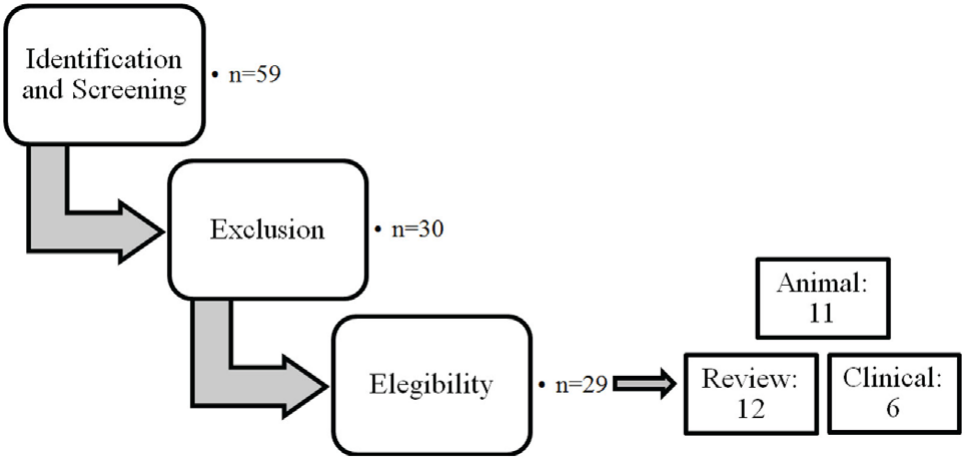
Schematic representation of the selection procedure for the articles included.

The different steps of the literature selection procedure are shown in Figure 1. An initial search resulted in 59 retrieved papers. From this total, 30 articles were excluded after revision of the contents based on the eligibility criteria described above. Twenty-nine articles were considered relevant for inclusion. Of these 29 publications, 11 reports were categorized as animal studies (Table 2), 6 as clinical studies (Table 3), and 12 as review papers (Table 4).

**Table 2:** Animal studies on bone healing in type 2 diabetes mellitus (T2DM) published between January 2007 and March 2017 (in descending order).

Reference	Study objective	Animal model and type of T2DM induction	Type of bone healing/regeneration	Outcome
Wallner et al. (15)	Compare different stages of bone regeneration between diabetic and non-diabetic mice and evaluate the efficacy of FGF-9 and vascular endothelial growth factor A (VEGF-A) in bone repair	C57Bl/6J mice (Lepr mutation db/db, spontaneous diabetes)	Unicortical bone defect (Tibia)	T2DM affects bone regeneration, through impairment of osteoclastogenesis and decreased biomarker levels in diabetic mice such as runt-related transcription factor 2, PCNA, and osteocalcin. Impairment of angiogenesis and osteogenesis could be reversed by local application of FGF-9 and VEGF-A, the latter to a lesser degree
Brown et al. (5)	Study of the impact of T2DM on fracture healing	C57Bl/6J mice (diabetic-induced obese)	Tibia fracture model	Increased callus adiposity and likely a fate shift of mesenchymal stem cells (MSCs) toward the adipogenic lineage, could be involved in the observed weakened biomechanical properties and delayed fracture healing of diabetic bone
Chen and Wang (16)	Observe change of FGF-2 and IGF-1 serum levels post-fracture and explore its mechanisms during healing	Sprague-Dawley rats [diet-induced obese (DIO) + streptozocin IP injection]	Tibia fracture model	Possible synergistic effects and decreased levels of FGF-2 and IGF-1 during fracture healing are accountable for impaired bone regeneration and delayed union in diabetic rats
Fontaine et al. (17)	Evaluate the macrophage inflammatory protein 1 (MIP-1) and VEGF expression in a diabetic rat model of fracture healing	Zucker diabetic fatty (ZDF) rats (Lepr mutation fa/fa, spontaneous diabetes)	Femoral fracture model	Biomarkers expression highly differs between diabetic and non-diabetic conditions during fracture repair. The increased level of MIP-1 can be associated with the likelihood of delayed healing
Hamann et al. (18)	Assess the effect of the parathyroid hormone (PTH) on skeletal and metabolic function in diabetic fracture healing	ZDF fatty rats (Lepr mutation fa/fa, spontaneous diabetes)	Femoral fracture model	Increased bone formation, increased bone strength, and improved defect regeneration suggest that PTH partially reverses the detrimental effects of T2DM on bone
Ro˝szer et al. (9)	Assess the role of leptin in postnatal regenerative osteogenesis in diabetic mice	C57Bl/6J mice (Lepr mutation db/db, spontaneous diabetes)	Femoral fracture model	Deficiencies in leptin can be linked to compromised bone acquisition and regeneration capacity, through delayed periosteal mesenchymatic osteogenesis, premature apoptosis of cartilage callus, and impaired microvascularization
Hamann et al. (10)	Assess the impact of diabetes on the structural and cellular properties of bone	ZDF fatty rats (Lepr mutation fa/fa, spontaneous diabetes)	Subcritical femoral defect model	Reduced ALP activity and mineralized matrix formation, suggesting osteoblast differentiation impairment and having an impact on bone mass and bone regeneration. Subcritical bone defect in diabetic rats demonstrated delayed healing in T2DM conditions
Jeyabalan et al. (19)	Determine if the antidiabetic drug metformin shows adverse effects on bone mass and/or fracture healing	Wistar rats (n/a)	Femoral fracture model	Bone mass and bone healing do not seem to be affected by metformin in rats. No differences in bone resorption, cortical and trabecular architecture, fracture callus volume and mineral content were found, compared to saline-treated controls
Liu et al. (20)	Examine the potential side effects of rosiglitazone on bone formation in diabetic mice	A^vy^/a mice (spontaneous diabetes)	Distraction osteogenesis (Tibia)	Impact of diabetes on bone healing after distraction osteogenesis is unclear. Rosiglitazone decreased intramembranous endosteal bone formation and increased adipogenesis in the distraction gap of both diabetic and non-diabetic mice
Waddington et al. (21)	Characterize biomarkers for oxidative stress and primary antioxidant enzymes during fracture healing in diabetic conditions	Goto-Kakizaki rats (spontaneous diabetes)	Mandibular implants	Delayed bone healing can be related to the absence of catalase enzyme activity, diminished by the affected oxidative environment due to hyperglycemia
Xu et al. (22)	Determine the possible relationship between peroxisome proliferator-activated receptor gamma (PPARγ) and core-binding factor α1 (Cbfα-1) in T2DM bone repair	Sprague-Dawley rats (DIO + streptozocin IP injection)	Distraction osteogenesis (Tibia)	Impaired fracture healing in T2DM rats may be caused by the increased expression of PPARγ mRNA and decreased levels of CBFα-1 mRNA in the bone marrow

**Table 3:** Clinical studies on bone healing in type 2 diabetes mellitus published between January 2007 and March 2017 (in descending order).

Reference	Main objective	No. of patients	Outcome
Hernigou et al. (23)	Mitigate wound infection and promote non-union healing in diabetic patients by percutaneous injection of bone marrow mesenchymal stem cells (BM-MSCs)	172	Treatment with BM-MSCs increased fracture healing in 82% of the diabetic patients, compared to 62% of diabetic patients treated with standard bone iliac crest autograft
Wukich et al. (24)	Compare the outcomes of retrograde intramedullary nailing for tibiotalocalcaneal arthrodesis (TTCA) in patients with and without diabetes	117	Despite an increased rate of superficial infections, retrograde intramedullary nailing proved to provide a high likelihood of successful limb salvage with TTCA in diabetic patients, similar to the outcomes of non-diabetic patients
Nozaka et al. (25)	Evaluate the progression of ankle fracture healing in a diabetic patient after the use of an Ilizarov ring fixator	1	Patients suffering from diabetes experience difficulties during fracture healing with increased possibility of non-union. In cases of Charcot arthropathy in which the fragment diameter is very small, it is more suitable to use an Ilizarov ring fixator instead of internal fixation
Ricci et al. (26)	Identify the risk factors for failure of lock plate fixation of distal femur fractures	326	It was determined that, along with open fracture, diabetes mellitus (DM) was an independent risk factor for reoperation to promote union and deep infection
Shibuya et al. (27)	Determine the risk factors associated with non-union, delayed union and mal-union in diabetic patients after foot and ankle surgery	165	It was determined that surgery duration, hemoglobin A1c levels >7% and especially peripheral neuropathy are statistically significantly associated with bone healing complications
Kline et al. (28)	Observe the rate of infection, rate of surgical complications, and the rate of non-union/delayed union in DM vs non-diabetes during tibial pilon fracture repair	81	In diabetic patients, the rate of infection was 71% (43% deep infection), and the rate of non-union/delayed union was 43%, in comparison with 19% (9% deep infection) and a 16%, respectively, for non-diabetic patients. This demonstrates that the presence of diabetes elevates the risks of complications during the management of tibial pilon fractures

**Table 4:** Review articles on bone healing in type 2 diabetes mellitus (T2DM) published between January 2007 and March 2017 (in descending order).

Reference	Article’s focus
Bahney et al. (29)	Review recent insights into the role of vascularization during the fracture healing process and highlight the need for an update in the endochondral repair model to promote adequate bone healing
Hayes and Coleman (30)	Revision of the literature supporting the application of MSCs in fracture repair in diabetic conditions and the possible causes promoting the dysfunction of the bone fracture healing process
Jiao et al. (31)	Study of different aspects that have been shown to impact bone and the skeletal repair process in T2DM, such as inflammation, reactive oxygen species formation, advanced glycation end products, hyperglycemia, especially in osteoblast differentiation and cellular bone turnover
Dede et al. (32)	Study the causes involved in the promotion of fracture risk in patients with T2DM, and discussion of the influence of reported research outcomes such as higher BMD, AGEs accumulation, and suppression of bone turnover under diabetic conditions on fracture risk
Fadini et al. (33)	Revision of the physiological and molecular bone marrow abnormalities associated with diabetes and also representing a potential root for the development of multiorgan failure characteristic of advanced diabetes
Razzouk and Sarkis (34)	Description of the impact of epigenetics on diabetes mellitus and smoking, and their significance in bone repair
Sathyendra and Dorowich (35)	Discussion of the factors influencing bone healing, such as diabetes, and the biology involved in the regeneration of new bone after fracture
Borrelli et al. (36)	Revision of the negative influence that certain clinical conditions, such as chronic inflammation, diabetes, aging, etc., exert on bone repair after fracture
Claes et al. (37)	Study the main factors promoting fracture healing impairment, with a particular emphasis on the role of inflammation
Simpson et al. (38)	Investigate the effect exerted by the main classes of diabetic drugs on the skeletal system, with special focus on fracture healing
Roszer (8)	Summarize the most recent reports supporting the idea that inflammatory signaling increases chondrocyte and osteoblast death and prolongs osteoclast survival, resulting in impaired bone regeneration in diabetic conditions
Retzepi and Donos (39)	Discuss the clinical evidence supporting a higher rate of complications during fracture healing in diabetic patients and provide a synthesis of the possible molecular mechanisms that are part of the diabetic bone healing pathophysiology

## Bone Tissue in the T2DM Environment

### Cellular and Molecular Composition of the Diabetic Bone

A good way to understand the pathophysiological effects of T2DM on bone is to study the composition of bone tissue at cellular and molecular level. Older studies have reported a high level of non-enzymatic cross-linking of the collagenous matrix, increasing the presence of AGEs that cause a lessened bone strength (40, 41). Collagen, in particular collagen type I, is a crucial protein in the maintenance of bone biomechanical strength due to its capability to generate intermolecular cross-links with adjacent collagen molecules. It has been shown that excessive non-enzymatic cross-linking hampers osteoblastic activity and this possibly through the interaction with the cell surface receptor of AGEs (RAGE) (13, 42), which decreases the synthesis of type I collagen, thus making collagen fibers brittle and accumulating excess microdamage. For this reason, AGEs are used as biomarkers for the assessment of increased risk of fractures (39).

Recently, a study from our group evaluated the levels of AGEs in bone from diet-induced obese (DIO) C57Bl/6 mice, under high-fat diet (HFD) treatment (43). By means of Raman spectroscopy, ratios were calculated using the Raman-specific bands for the AGEs pentosidine (~1,495 cm^−1^) and carboxymethyl-lysine (CML; ~1,150 cm^−1^) and normalizing each to the CH_2_ band (1,450 cm^−1^), which represents the organic matrix of bone. It was observed that the cortical area of femora from DIO mice presented significant accumulation of AGEs when compared to age-matched lean control mice, confirming the excessive generation of these species in bone under T2DM conditions (43). Furthermore, combined micro-computed tomography (microCT) analysis, three-point bending tests and finite element modeling revealed that DIO mice had reduced bone strength and structural stiffness, and increased material stiffness associated with the accumulation of AGEs in the bone tissue under T2DM conditions (43).

Evidence has also been pointed toward a detrimental effect of T2DM-induced hyperglycemia on osteoprogenitor cells. In a study from Hamann et al. (10), osteoblast activity was found impaired in a model of Zucker diabetic fatty (ZDF) rats (10). Although there seemed to be a similar supply of osteoblastic precursors between the diabetic and non-diabetic rats (determined by colony-forming assays), T2DM conditions impaired osteoblast differentiation based on a 55% lower mineralized matrix formation after 21 days of cell differentiation (10). More recently, it was reported that the mineralization capacity and the alkaline phosphatase activity of bone marrow stromal cells (BMSCs) derived from rats under T2DM conditions was significantly decreased compared to controls. This impairment in osteogenic potential was explained by a significant reduction in reduced gene expression levels of β-catenin, cyclin D1, and c-myc, thus inhibiting the Wnt signaling pathway (44).

### Biomarkers of Skeletal Dynamics in T2DM

According to the study from Reyes-García et al., the parathyroid hormone (PTH) can be linked to bone resorption in T2DM due to its positive association with the markers serum tartrate-resistant acid phosphatase-5b in T2DM patients, and serum terminal cross-linked telopeptide of type-I collagen (s-CTX) (45). The latter showed decreased levels in T2DM conditions according to several recent studies, along with the bone formation marker P1NP which was also found decreased (46, 47). Serum levels of TRAP and bone formation marker osteocalcin (OC) have been found diminished in T2DM patients, contrary to high serum levels of sclerostin (a potent inhibitor of bone formation) (48, 49), suggesting that individuals with T2DM present a reduced bone turnover. These data were supported by a recent study from our group, in which s-CTX and s-OC levels were found decreased in DIO mice, compared to age-matched lean controls (50).

It is important to highlight that there are conflicting results reported in existing literature for several of these biomarkers. Some studies report increased CTX levels, either unchanged or increased levels of OC (51, 52) and increased P1NP (53) in T2DM patients compared to healthy individuals. Differences in metabolic status, duration of the diabetic condition, and diabetic medication treatment being used by the time of the measurements might be responsible for these contradictions (11). Moreover, inconsistent results in the bone turnover process under T2DM conditions are also reported for rodent models in literature. T2DM models of rats have shown decrease bone formation with increased bone resorption (18, 54) in contrast to other studies showing lower levels of these parameters (55). Likewise, studies in T2DM mice models have showed different outcomes, with increased bone resorption (56) coupled with decreased or unchanged formation parameters (57), in contrast to decreased levels of bone resorption and formation (2, 50) and even reports showing increased bone turnover with higher levels of both resorption and formation in the T2DM group (3). These contradictory results may also be explained by the duration of the HFD treatment and the disease, the own response of each species’ strains to the T2DM effects, or by the different levels of expression of skeletal growth modulators such as PTH and IGF-1, which have been associated with the bone resorption and/or bone formation processes (58, 59).

### Vascularization of Bone under T2DM Conditions

Blood supply is critical for the development and proper functionality of bone tissue, providing oxygen, nutrients, and minerals essential in bone regeneration (60). Many studies have addressed and demonstrated the key role of the vasculature and angiogenesis in fracture repair, evaluating the contribution of elements such as endothelial progenitor cells (EPCs), ischemia, and proangiogenic factors such as vascular endothelial growth factor (VEGF) and HIF1α (61–63).

It is known that T2DM is associated with several vascular complications including diabetic neuropathy, nephropathy, retinopathy, peripheral vascular disease, ischemic heart disease, among others (64–68). Arterial medial calcification is caused by the biomineralization of vascular cells, impairing the arterial vessel system and thus the functionality of the vasculature (67). Several findings have tried to explain this phenomenon, including the identification of upregulated bone alkaline phosphatase, a known modulator of mineralization (69), helped by elastin degradation in blood vessels undergoing arterial medial calcification (70). Another possible mechanism for arterial stiffening is the role of the transcription factor Msx2 in vascular mineralization. A model of Msx1 and Msx2 gene deletion in obese HFD-fed LDLR(−/−) mice showed that decreased levels of these transcription factors (34 and 95%, respectively) resulted in reduced expression of Wnt genes and aortic osteogenic progenitors, such as Shh and Sca1, thus limiting the osteogenic differentiation and mineralization potential of cells involved in vascular calcification (71). Moreover, studies have shown that AGEs and RAGE seem to play a role in vascular calcification, which implicates further potential complications for T2DM patients. A recent study from Koike et al. assessed the effects of AGEs on the rat vascular smooth muscle cell (VSMC) line A7r5 *in vitro*. Cells incubated with AGEs in calcification medium exhibited increased calcium deposition compared to bovine serum albumin cells (control). Visualization and quantification of significantly increased VMSCs apoptosis after treatment with AGEs was possible by means of TUNEL and Hoechst stainings, showing an AGE-induced apoptosis of 83% compared to 1% in controls (72). Moreover, mRNA expression of the NAD(P)H components Nox1, Nox4, and p22^phos^ was significantly upregulated in AGEs treated VSMCs, and when these components were silenced after siRNA transfection, AGE-induced apoptosis was markedly reduced (42–47%). These data suggest that the activation of NAD(P)H oxidase regulates the AGE-induced apoptosis of VSMCs (72). In addition to this, another study explored the mechanism of AGE-induced diabetic calcification using also the same cell line A7r5 (73). In this case, the VSMCs also showed increased levels of AGE-induced arterial calcification, and the serum level of the AGEs species CML was positively correlated with calcium content in the arterial walls. Furthermore, the CML/RAGE signal intensity seemed to increase with the diabetes-induced vascular calcification progression, and when the calcification pathway was blocked by using anti-RAGE antibodies, the calcium deposition and ALP activity were significantly reduced by approximately 50% (73). This confirms results from previous studies suggesting that AGEs accumulation is detrimental for vascularization and thus for bone tissue health in T2DM conditions.

In addition to the T2DM-induced vascular calcification, the vascular progenitor cells seem to be affected by the metabolic disease. EPCs are known for their expression of endothelial markers (VEGF, CD34) and enhancement of angiogenesis after differentiating into mature endothelial cells (74), thus aiding in wound healing and tissue regeneration with their proangiogenic capacities. In a study from Lombardo et al., analysis of different subpopulations of EPCs and circulating endothelial cells in peripheral blood was carried out on patients suffering from T2DM and healthy controls. It was found that T2DM individuals showed an increased number of highly immature EPCs (pre-EPCs) expressing early hematopoietic markers CD117 and CD133 (74). Levels of EPCs (coexpressing CD34/VEGF/CD133) and highly differentiated EPCs, or “late-EPCs” (coexpressing VE-cadhering and CD31) were also assessed. Results showed that there was no significant difference between EPCs level of T2DM and control groups. On the other hand, a highly significant decrease of late-EPCs level was found in T2DM individuals (74).

All this sets a precedent of the state in which bone tissue under T2DM conditions may be before a fracture event occurs.

## Bone Fracture Healing in Normal Conditions

When bone tissue gets damaged, the fracture healing process is successfully completed when the proper biological and mechanical conditions for tissue repair exist and the surrounding microenvironment, i.e., the host bed, is not compromised. This process undergoes a sequence of biological events that have been clearly described and divided by Claes et al. (37) into three phases: inflammation, repair, and remodeling. The description of these phases has been based on the well-studied rat fracture healing model (75). The process is similar for humans and for other larger animal models, taking place over longer periods of time.

In the inflammatory phase, vasodilatation and exudation of plasma and leukocytes occur at the site of the lesion after the rupture of blood vessels and damage of the bone and surrounding tissues. Then, a fracture hematoma is formed, characterized by the presence of different inflammation-related cells, such as macrophages, leukocytes, and cytokines. Among the cytokines are interleukin 1 (IL-1) and 6 (IL-6), TNF, members of the transforming growth factor beta (TGF-β) superfamily like bone morphogenic protein 2 (BMP-2) and 6 (BMP-6), and angiogenic factors such as VEGF. Stimulation of the angiogenesis process occurs, and newly formed blood vessels provide access to osteoprogenitor cells that will contribute to fracture repair. Next, the hematoma gets progressively replaced by a granulation tissue containing collagen, produced by fibroblasts, cells, and new capillaries (37).

The second phase is the repair phase. The anabolic mechanism for the periosteal callus formation is endochondral ossification, with the primary development of a soft cartilaginous callus succeeded by its transformation into a hard bony callus, usually temporally overlapping with the inflammatory phase (76). After 10–14 days of chondrocyte proliferation in fracture healing experiment involving rats, cell hypertrophy, calcium release, and subsequent apoptosis have been observed (77). Once the cartilaginous callus segments successfully bridge the fracture, blood vessels are allowed to occupy the calcified cartilage area and promote hypervascularization due to reduced interfragmentary movement and tissue strain during loading of the fracture. Hypervascularization in turn stimulates the recruitment of monocytes and mesenchymal stem cells (MSCs), which will differentiate into osteoclasts and osteoblasts, respectively; the former resorbing the calcified cartilage and the latter generating new bone tissue into the resorbed lacunae, ultimately leading to the formation of woven bone with a trabecular structure (37).

Finally, after the fracture gap has been filled with new woven bone, osteoclastic activity takes place at the outer surface to start the resorption of the periosteal callus and therefore the onset of the remodeling phase. The previously formed woven bone tissue gets transformed into lamellar bone through osteon formation, and the remodeling and resorption of the periosteal and medullary calluses conclude with the successful reshaping of a diaphyseal bone, a process that can take between 5 and 8 weeks in rats and years in humans. Some characteristics of this phase are the diminishing of the vascularization process to pre-fracture levels and the reduction of inflammatory cytokine levels, with the exception of IL-1, TNF, and BMP-2, which can still be found to be highly expressed (37).

## Bone Fracture Healing Under T2DM Conditions

The result of the normal fracture healing process is the obtainment of a fully loadable and reconstructed bone. However, in the presence of hyperglycemia, bone tissue experiences alterations in quality, composition, and biomechanical properties and these can lead to fracture healing impairment or even non-union (21, 37, 78).

**Figure 2:**
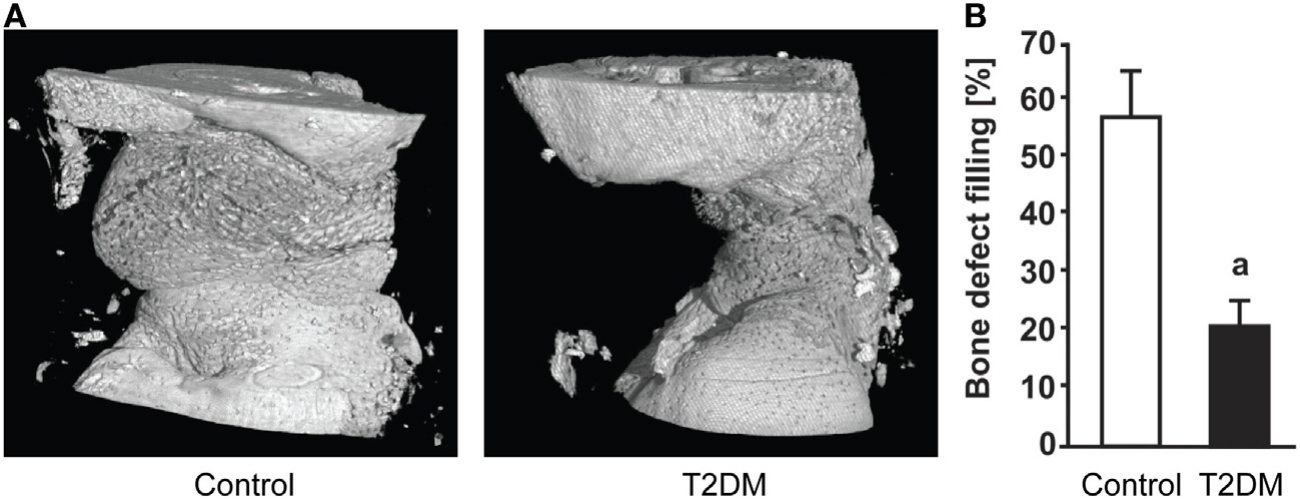
**(A)** 3D representation generated after micro-computed tomography (microCT) scanning of the subcritical femoral defect model in control vs type 2 diabetes mellitus (T2DM) rats, 12 weeks post-surgery. **(B)** microCT-based quantification of the bone defect filling in control and T2DM femora, 12 weeks post-surgery. *n* = 7–10. ^a^*p* < 0.01. Figure taken and adapted from Ref. (10).

**Figure 3:**
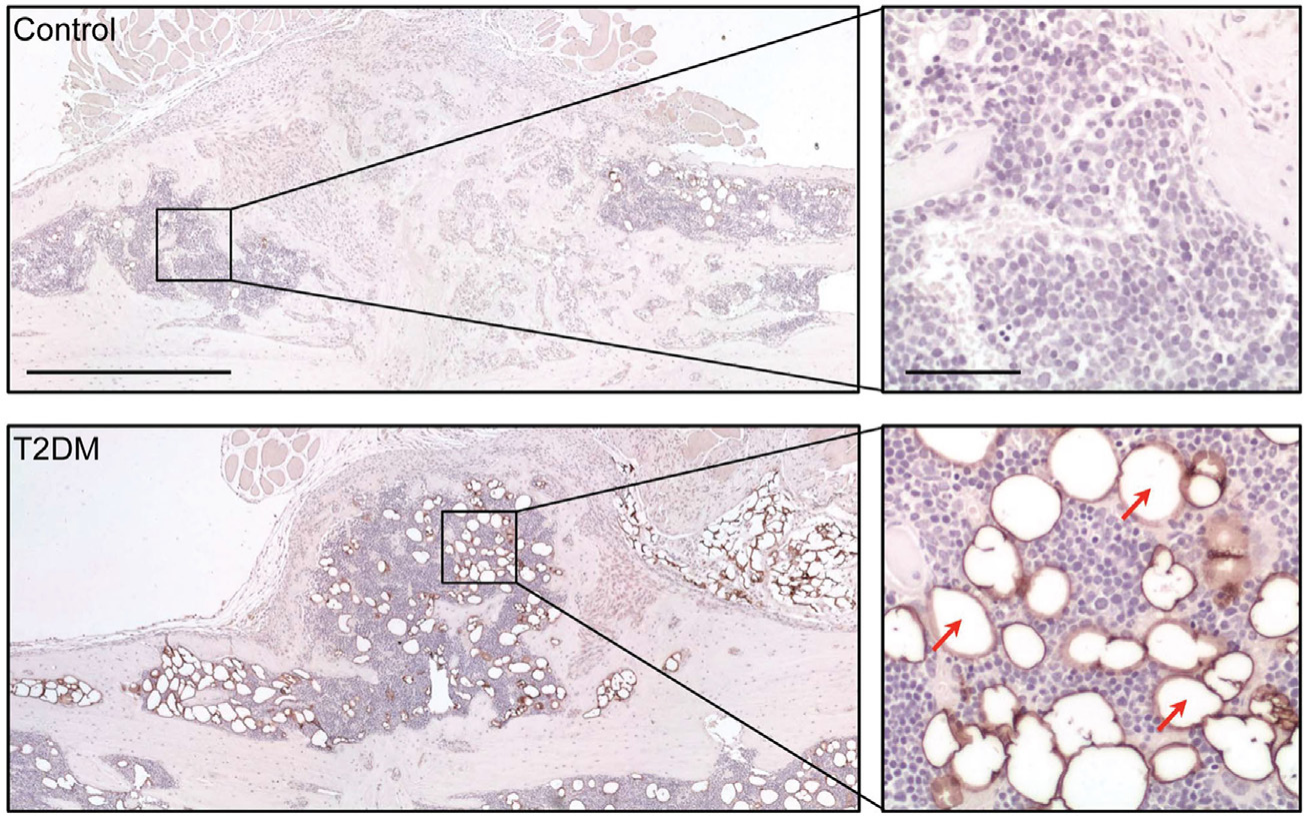
Assessment of adipocyte presence in the fracture callus of control and type 2 diabetes mellitus (T2DM) mice tibiae through immunological staining for peripilin. Right panels show a magnified area of the sections, where stained adipocytes (red arrows) can be appreciated more clearly. Timepoint post-fracture surgery: 21 days. Black scale bar in left panel = 1 mm. Black scale bar in right panel = 100 µm. Figure taken and adapted from Ref. (5).

Imaging techniques such as micro- and nanoCT stand as a powerful tool to accurately assess the progression of bone repair (Figure 2). In 2014, a study from Brown et al. using C57BL/6 T2DM-induced mice demonstrated delayed fracture healing, increased callus adiposity, and hampered biomechanical properties in T2DM conditions. Using microCT scanning, the authors were able to observe that there was a trend toward decreased callus vascular volume and a significant decrease in fracture callus bone volume at day 21 after fracture in HFD-fed mice compared to control mice. Furthermore, delay in reaching peak bone volume and a significant decrease in woven bone area was found at day 28 post-fracture in HFD-fed mice. The authors also discussed the presence of increased adiposity found in fracture calluses of HFD-fed mice only (Figure 3). It is proposed that the balance of MSCs differentiation toward osteoblast and adipocyte lineage is altered to favor the latter. It is further discussed that the upregulated levels of PPARγ found in the fracture calluses of HFD-fed mice are indeed most likely promoting the MSCs fate switch. Finally, it was determined that during the entire fracture healing process the osteoclast phenotype remained within normal parameters (5). This was also confirmed in a study using ZDF rats where osteoclast function did not show differences between diabetic and control animals (10). The observed abnormality of increased PPARγ expression and elevated marrow adiposity in the femoral diaphyseal area has been confirmed by other studies (3, 22), using the DIO C57BL/6J mice model under HFD treatment and comparing to low-fat diet-fed mice (3), confirming the T2DM fracture healing impairment cocaused by the event of fate switch from osteoblasts to adipocytes.

**Figure 4:**
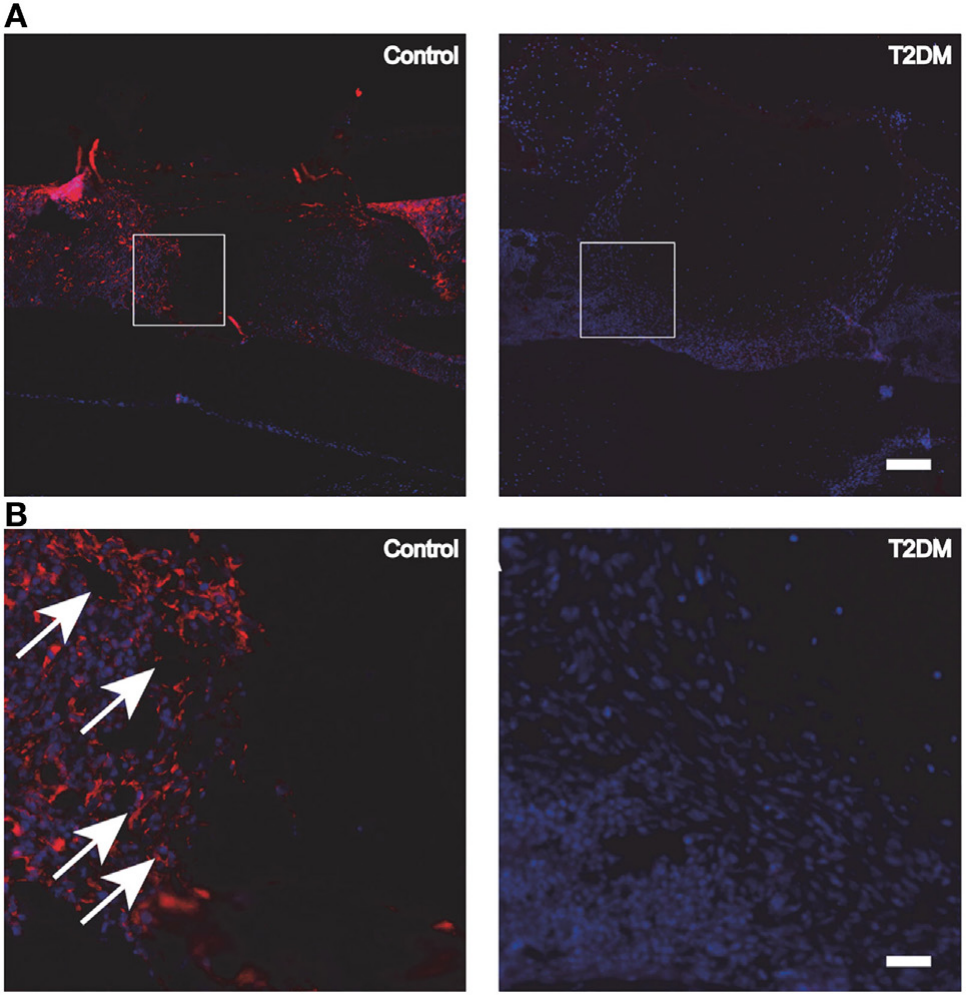
**(A)** Fluorescence immunohistochemistry staining for PECAM-1, to detect the presence of blood vessels and endothelial cells in control vs type 2 diabetes mellitus (T2DM) mice tibiae, 3 days post-surgery. **(B)** Magnification of the areas in **(A)** represented by white squares. White arrows signal blood vessels and endothelial cells, stained in red. White scale bar in **(A)**: 200 µm. White scale bar in **(B)**: 45 µm. Figure taken and adapted from Ref. (15).

The decreased capability of MSCs committing to osteoblastic differentiation is further evidenced by the inhibition of expression of transcription factors, crucial for the development of the osteoblastic phenotype such as Dlx5 and runt-related transcription factor 2 (Runx-2), during intramembranous bone healing in a marrow ablation model (79). Moreover, reduced immunohistochemical indices (up to 50%) of cell proliferation rate in the diabetic callus further support the decreased number of osteoblasts as part of the mechanism for impaired bone healing (80). In addition, protein expression levels of several growth factors closely related to osseous healing such as PDGF, IGF-1, VEFG, and TGF-β1 have been reported to decrease in diabetic rodents models (80, 81). Particularly for VEGF, several studies report alterations in its expression levels during the fracture healing process under T2DM. Rőszer et al. (9) studied the femoral fracture healing model in leptin receptor-deficient (db/db) T2DM mice. After 7 and 14 days post-surgery, the authors found a low VEGF expression in the plasma and callus tissue of the db/db mice, compared to lean control mice. In addition, deficient microvascular invasion and elevated chondrocyte apoptosis in the fracture callus of the db/db mice were shown, suggesting a compromised cartilage-to-bone transition and explaining the delayed bone healing in these mice (9). Impaired angiogenesis was also observed in another study using the db/db T2DM mouse model to assess the tibial fracture healing process (Figure 4). An additional study involving femoral fracture models using ZDF rats (17) also demonstrated a significant decrease in the serum levels of VEGF at different timepoints during the early stages of the fracture healing process (2 weeks). In addition, the expression levels of macrophage inflammatory protein 1α, known to be highly involved in osteoclastogenesis, were found increased in the serum of the rats with T2DM. This may be potentially extending osteoclast activity during bone repair and compromising the healing process (17).

Nevertheless, the effect that T2DM causes to osteoclasts remains controversial in literature. An *in vitro* study evaluating the murine monocytic cell line RAW264.7 reported that high concentrations of D(+)Glucose (25 mM) inhibited TRAP activity and osteoclast differentiation induced by RANKL (a key factor in osteoclastogenesis) (82). In contrast, other studies involving circulating osteoprogenitor precursors (human) and BMSCs (mice) have reported an increase in osteoclast and osteoclast precursor numbers by TRAP staining, and increased expression of the osteoclast-specific biomarker cathepsin K (46, 83). Nonetheless, when the *in vivo* bone fracture healing model is assessed the data suggest no changes in the osteoclast compartment. Several fracture healing studies in mice and rats agree with the fact that osteoclast number and functionality remain unchanged during bone repair under T2DM conditions, in terms of TRAP-staining positive cells and TRAP mRNA expression levels (5, 10). It is possible that the stress caused by the fracture event in the *in vivo* T2DM microenvironment alters the mechanisms governing osteoclast functionality.

Biomarkers of bone formation and resorption during the fracture healing process have also been evaluated. A study from Chen and Wang (16) showed that serum and fracture callus levels of FGF-2 and IGF-1 were significantly decreased in a tibial fracture healing model of diabetic rats. It was also noted that rats with DM presented less density and area of newly formed bone at the fractured ends of the tibiae (16). The study by Wallner et al. (15) characterized the bone regeneration process at different timepoints by means of a unicortical bone defect model applied to T2DM and control mice. Immunohistochemical stainings for OC and RUNX-2 were performed at days 3, 5, and 7 post-surgery, revealing a significant decrease in the levels of both biomarkers in T2DM mice, specifically at days 3 and 7 post-surgery for RUNX-2 and OC, respectively. It can be speculated that the decreased OC level in T2DM mice is caused by the decreased RUNX-2 levels at the early stage of the fracture healing process, since RUNX-2 is known for regulating OC expression (15). Moreover, Hamann and coworkers published two studies (2011 and 2014) where levels of OC were measured in a critical-sized (10) and in a subcritical-sized (18) bone defect model in T2DM rats. In both studies, OC levels were diminished, by 40% (10) and 52% (18), respectively. Serum levels of the biomarker CTX were also measured, and in both studies these were found to be increased by threefold in T2DM rats, compared to healthy controls (10, 18). In addition, TRAP levels were increased by 70% in diabetic rats (18). The results from these studies confirm diminished bone formation and increased bone resorption in T2DM bone fracture healing. Taken together, these findings on biomarker tracking during skeletal tissue repair support the idea that bone healing and regeneration is impaired by T2DM, particularly *via* the alteration of the bone turnover process.

The inflammatory phase of the fracture healing process is crucial for providing oxygen, nutrients, and the osteoprogenitor cells necessary for the bone repair (37). It is known that T2DM favors an increased inflammatory state, which alters several factors involved in the mechanisms of bone healing and promotes the activation of inflammatory mediators, such as reactive oxygen species (ROS) and AGEs (8). One key mediator present in the early inflammatory response after bone fracture which starts the bone repair process is TNF-α. This inflammation-related factor and the members of the TNF-α receptor family have been identified to play a key role in the initiation of apoptosis (8). Bone fracture healing models of T1DM CD-1 mice have been used for the assessment of the effects of TNF-α on the bone repair process. It was reported that TNF-α triggers the expression and activity of proapoptotic factors such as caspase-3, -8, and -9, inducing chondrocyte apoptosis, resulting in reduced callus and cartilage area (84). Furthermore, TNF-α contributes to the endothelial cell proliferation impairment, reduced tube formation, and suppressed VEGF expression in fractured tibiae and femora of mice, compromising the angiogenesis of the healing process (85). The effect of TNF-α on bone was mediated by FOXO1, a transcription factor involved in the expression of the proapoptotic factors p21 and caspase-3 (85). Even though these biological events have not been explored in bone fracture healing of a T2DM animal model, the findings in the fractured bone of T1DM mice set a precedent for what may be happening in the former. This idea is supported by a study from Halade et al. (86), which showed increased gene expression of TNF-α in femora from obese, hyperglycemic, and insulin-resistant mice, fed with a corn oil-enriched diet (86).

## Clinical Complications of Diabetes in Bone Surgery

Diabetes mellitus has been classified as a risk factor for failure in operative procedures for bone fractures due to post-surgery complications (26). Patients suffering from DM that need to undergo fracture surgery have shown an increased rate of complications after the procedure has been carried out (25, 87). In 2009, a study including 81 patients (14 fractures in 13 individuals with DM, 69 fractures in 68 non-DM individuals) who received primary treatment for a tibial pilon fracture, determined the rate of infection, the rate of delayed union, non-union, and rate of surgical wound complication after the procedure (28). Though the rate of surgical wound complication presented no differences (7% for both groups of individuals), the rate of infection in patients suffering from DM was as high as 71% (43% for deep infections), compared to 19% for patients without DM (9% deep infection). Furthermore, the rate of delayed union/non-union was reported to be 43% for DM patients, against a 16% for controls (28). A study from Wukich et al. (24) also showed a higher rate of superficial infection in patients with DM, although it was shown at the same time that the overall incidence of complications between patients with and without DM was not significantly different (24). Another study included 165 patients suffering from DM that underwent arthrodesis, osteotomy, or fracture reduction. The objective was to determine which diabetes-related comorbidities, including tobacco use, peripheral vascular disease, peripheral neuropathy, among others, were positively associated with fracture delayed union, non-union, and mal-union after foot and/or ankle surgery (27). After bivariate analyses and covariates adjustments, results showed that the surgery duration, HbA1c levels >7% and particularly peripheral neuropathy were predictors for bone healing impairment in individuals with DM after surgery (27). Specifically for ankle fracture, it is known that around 30% of patients with DM do not regain complete functionality after treatment, compared to only 10% of incomplete functional recovery in cases without DM (88, 89). Amputation is another concern among patients with DM after ankle fracture. A fourfold to fivefold increased rate of amputation after fracture treatment has been reported in patients with DM in comparison to controls (90–92). In the case of open ankle fractures, this rate increases even more, up to an alarming 42% (93).

Regarding hip fractures, they are among the most common orthopedic fractures, having a yearly incidence as high as 1% in the United States (94, 95), and they are likely to increase also in the UK in the next 15 years (96). T2DM is known to increase this incidence rate by 1.4- to 1.8-fold (97, 98) due to mechanisms still not fully understood (99, 100). It has been reported that T2DM decreases the strength of the femoral neck of rats by 64% when compared to healthy controls (101). As seen in patients with several other orthopedic fractures, T2DM is likely associated with complications after treatment of hip fracture, such as surgical site infection, pressure ulcers, cardiac post-operative complication, and increased rate of mortality (102–104).

## Effects of Antidiabetic Medication on the Homeostasis and Repair of the Diabetic Bone

One important factor to take into account when studying bone fracture healing in patients suffering from T2DM is their history of the use of antidiabetic medication. Some blood glucose-lowering treatments have been associated with alterations in skeletal properties (53, 105), which can be either beneficial or detrimental for the process of fracture healing.

Thiazolidinediones (TZDs), including pioglitazone, rosiglitazone, and troglitazone, are a family of synthetic PPARγ agonist drugs. TZDs are widely used as treatment for T2DM patients because of their effective improvement of insulin sensitivity, but have also been proven to act adversely against skeletal homeostasis (106, 107). The first clinical evidence of TZDs relation to bone fracture risk was reported in the “A Diabetes Outcome Progression Trial” studies (108, 109). The authors found that the increased risk of fracture manifested after 12 months of treatment, and the cumulative risk of fracture was 15.1% for rosiglitazone female T2DM patients, compared to 7.3 and 7.7% for metformin and glyburide females T2DM patients, respectively (109). Due to their nature as PPARγ agonists, it has been proposed that TZDs favor a preferential differentiation of bone marrow MSCs into the adipogenic lineage, decreasing commitment toward osteoblast differentiation, thus disrupting cellular homeostasis (110). *In vivo* experiments using primary human BMSCs revealed that TZDs reduced the number of osteoblast positive colonies and the RNA expression of osteogenesis markers Runx2 and OC, but the adipogenic differentiation was favored, after observing increased adipocytes colony-forming units and PPARγ RNA expression (111). Furthermore, a study from van Lierop et al. (53) assessed the effect of metformin on serum sclerostin and other bone biomarkers. Metformin is a popular antidiabetic oral drug used among T2DM patients, which has been associated with decreased fracture risk (100) and increased osteoblast differentiation by transactivation of RUNX2 (112). Compared to healthy, untreated control subjects, the authors found that male T2DM patients under TZDs treatment experienced an increase of 11 and 16.8% in s-sclerostin and s-CTX levels, respectively, contrary to the metformin-treated group, which did not show any significant changes in s-sclerostin but had a 19% decrease in s-CTX (53). Another study followed up for 15 years the treatment with metformin in a cohort at very high risk of developing diabetes. Results showed that the diabetes incidence was reduced 18% in metformin-treated subjects compared to the placebo group, and the cumulative incidences of diabetes were 56% for subjects receiving metformin and 62% for placebo subjects (113).

An antidiabetic medication that has also been reported to produce osteogenic effects is the glucagon-like peptide 1 receptor agonist (GLP-1RA). Studies involving ovariectomized rodent models have shown that after GLP-1RA treatment, trabecular bone mass and connectivity, s-OC and s-alkaline phosphatase levels, and osteoclast numbers are significantly increased, but osteoclast activity and s-CTX levels are decreased compared to untreated controls (114, 115). Furthermore, *in vitro* experiments showed that sclerostin expression was decreased in osteocyte-like MLO-Y4 cells under hyperglycemic conditions and GLP-1RA treatment (116). The authors confirmed the osteogenic effect *in vivo*, reporting decreased s-sclerostin levels, and increased BMD and s-OC levels in T2DM OLEFT rats treated with the antidiabetic medication (116).

The sodium-glucose cotransporter 2 (SGLT-2) inhibitors are another kind of drugs prescribed to T2DM patients. They control hyperglycemia by increasing the urinary glucose excretion, reduce body weight, and reduce body fat mass (117, 118). A recent study showed that, over the course of 104 weeks treatment, a 7.7% of T2DM patients under dapagliflozin (a commercially available SGLT-2 inhibitor) presented bone fractures, though the exact cause of this increase in fracture risk is rather uncertain (119). Other studies have reported increased levels of CTX and OC biomarkers (120), and an increase of serum phosphate levels (121, 122), in T2DM patients under treatment with SGLT-2 inhibitors. The latter findings may imply a potential upregulation of the PTH hormone (secondary hyperparathyroidism), triggering bone resorption and thus favoring fracture risk (105, 123).

The effects of additional antidiabetic medications, such as sulfonylureas and DPP-4 inhibitors, have been covered previously in literature, and together with the ones addressed in this article, are very well described in a review by Palermo et al. (124).

A topic that remains highly underexplored is the impact of antidiabetic drugs on bone fracture repair. A study from Liu et al. using a mouse model of T2DM (A^vy^/a mice strain) and distraction osteogenesis evaluated the effects of rosiglitazone on the bone repair process (20). After 2 weeks of osteotomy surgery, microCT analysis revealed that the distraction gap area occupied by new bone was reduced from 66 to 43% when untreated and rosiglitazone-treated A^vy^ mice were compared, respectively. In addition, histological and immunohistological assays determined that in the fracture site of rosiglitazone-treated mice, marrow fat presence was significantly increased, osteoprogenitors highly expressed adipocyte protein 2, and cell proliferation was compromised (20). Consequently, it is speculated that the use of TZDs is disadvantageous for the fracture repair process in T2DM patients. On the other hand, metformin was evaluated in another study using 3-month-old Wistar rats undergoing femoral osteotomy (19). The authors found no differences in cortical and trabecular thickness, trabecular bone volume and number, and periosteal and endosteal perimeter between the metformin-treated and untreated groups 4 weeks after fracture surgery. Taking all this into account, as more evidence is unveiled about the detrimental effects of many antidiabetic drugs on the skeletal system, the future direction of the line of research should be focused more on further proving the beneficial (or lack of) effects of metformin and other antidiabetic medications such as GLP-1RAs on bone tissue, so it can be taken fully into consideration when choosing the current safest alternative for T2DM treatment in bone fracture patients.

## Possible Therapeutic Strategies for Compromised Bone Repair Under T2DM Conditions

In the field of bone tissue engineering, MSCs represent a promising cell-based alternative against the challenges of treating bone defects, due to their self-renewal characteristics, the possibility to isolate them from many types of tissue and their ability to differentiate into multiple cell lineages (125). For instance, it was reported that treatment with autologous bone marrow MSCs was able to favor healing of non-union ankle fractures in patients suffering from DM (23). A great source of this type of cells is the periosteum, a structure covering the external surface of bone which is known to be crucial in the recruitment of osteoprogenitor cells during the events of bone regeneration and fracture repair (126–128).

Periosteum-derived cells (PDCs) have been used previously in tissue engineering approaches to study bone formation. By seeding human PDCs in calcium-phosphate scaffolds, it was possible to confirm the bone forming capacity of these constructs in an *in vivo* ectopic implantation model of NMRI^nu/nu^ mice (129). With this in mind, the potential regenerative power of PDCs look promising to be used in the form of tissue engineering constructs implanted in the diabetic microenvironment, to aid and enhance the bone healing process impaired by T2DM.

A recent study from Tevlin et al. (130) investigated the rescue of skeletal stem cells derived from the diabetic microenvironment to aid the bone fracture healing process. The authors discovered that, during femoral repair, the fracture-induced expansion of these diabetic stem cells was compromised by elevated levels of TNF-α, inhibiting in turn the expression of Indian hedgehog, and altering the expression of apoptosis-related and proliferation-related genes (130). Moreover, after local delivery of an Indian hedgehog-coated hydrogel into the fracture site of T2DM mice, mechanical strength tests showed improved bone strength, elevated proliferation, reduced apoptotic activity, and enhanced osteogenesis of the diabetic skeletal stem cells. These data demonstrated that the correction of progenitor cells derived from a compromised niche such as the diabetic microenvironment can be a potential therapeutic strategy for the rescue of the bone fracture healing process (130).

As previously discussed, impairment of vascularization and angiogenesis associated with T2DM greatly affect the bone healing process. Therefore, it is of utmost importance to find new approaches that can contribute to adequate vascular functionality in the aid of bone repair. Two studies evaluated the rescue of the functionality of impaired bone marrow-derived angiogenic cells (BMACs) from T2DM animals. Using the microRNA miR-27b mimic/inhibitor, Wang et al. (131) observed that, compared to controls, miR-27b mimic increased BMACs proliferation, adhesion, tube formation, delayed apoptosis, and suppressed expression of the anti-angiogenic protein semaphoring 6A and the pro-oxidant protein p66, which elevates mitochondrial ROS levels, thus increasing the angiogenic potential of the BMACs (131). Another study assessed the rescue of the angiogenic function of BMACs derived from T2DM animals *via* overexpression of adenoviral vector-mediated dominant negative Rac1, hampering functionality of endogenous Rac1, subunit of NADPH oxidase, which was found to have an increased activity in these cells (132). Results showed that tube number, tube length, adhesion, and migration ability were all seemed increased in the BMACs after treatment, compared to controls (132). Overall, recent relevant studies have been done on the rescue of the vascularization in the diabetic microenvironment, especially in the field of endothelial progenitors and proangiogenic cells. Nonetheless, research efforts should focus more on the impaired vascular system’s rescue during fracture healing under T2DM, as it remains as a much-underexplored topic. Much further experimentation is still needed to elucidate the detrimental effects of T2DM on vascularization during bone repair, for researchers to achieve an enhanced and successful fracture healing process under compromised conditions such as T2DM.

## Conclusion

**Figure 5:**
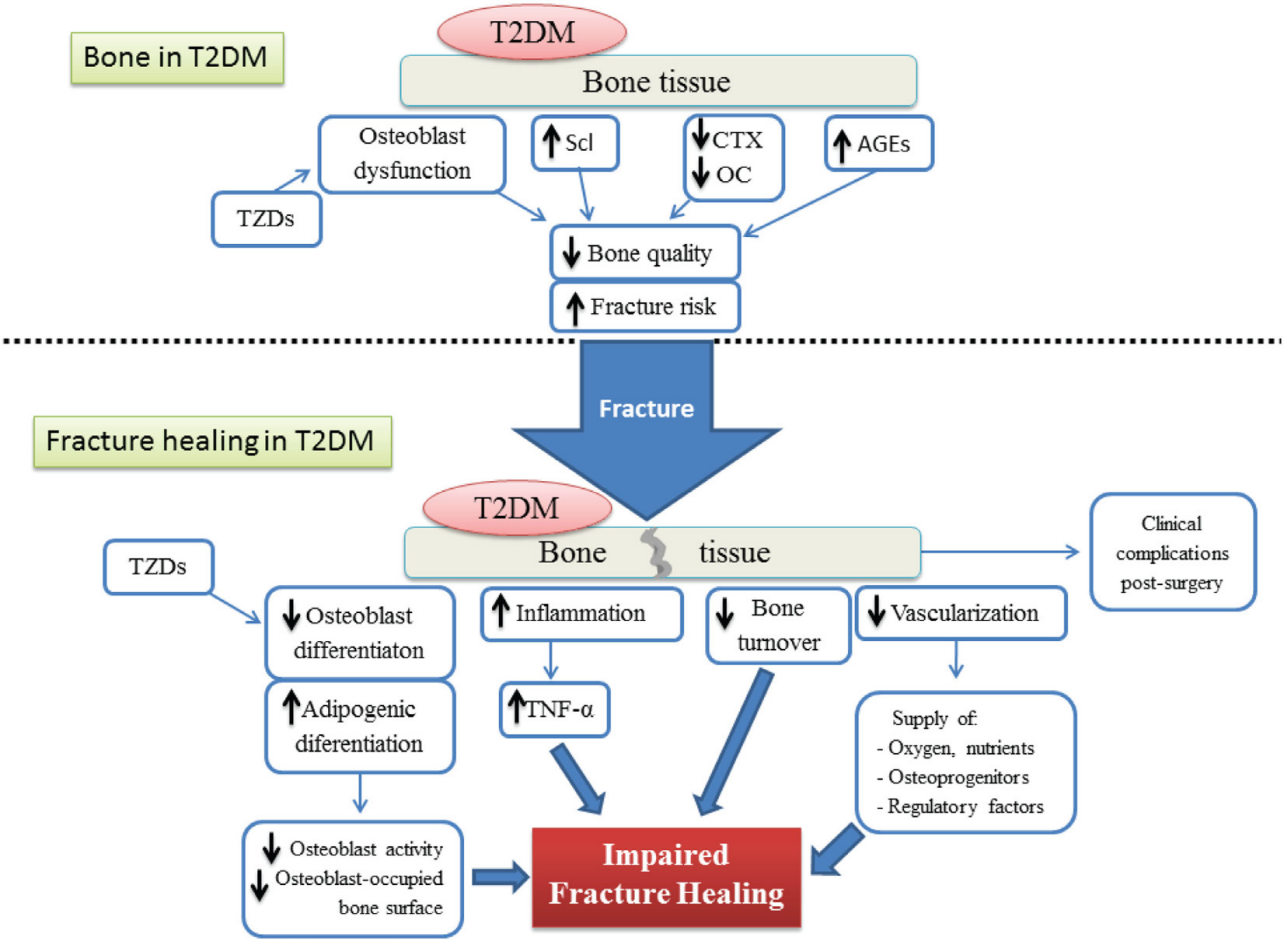
Schematic representation of both the bone tissue state and the different factors involved in the impairment of the fracture healing process, under type 2 diabetes conditions. T2DM, type 2 diabetes mellitus; AGEs, advanced glycation end products; TZDs, thiazolidinediones; Scl, sclerostin; CTX, terminal cross-linked telopeptide of type-I collagen; OC, osteocalcin; TNF-α, tumoral necrosis factor alpha.

The process of bone healing after fracture is highly compromised by different factors altered by T2DM (Figure 5). The detrimental effect of T2DM on the regenerative ability of bone seems to be acting at cellular, molecular, and biomechanical levels. Fate switch of MSCs favoring the adipogenic lineage causes a significant decrease in woven bone area and creates a negative impact on bone formation and cellular composition. On the other hand, an exacerbated presence of AGEs allows the formation of excessive non-enzymatic cross-linking, hampering type I collagen synthesis and promoting the brittle of collagen fibers which in turn generates a deficiency in biomechanical strength. Critical stages of the fracture healing process, such as the inflammatory phase and vascularization, are altered by T2DM and this affects the dynamics of the mechanisms involved in successful bone repair. The impact of T2DM on bone fracture healing represents an important problem for patients suffering from diabetes, since bone fractures could lead to delayed healing, non-union, and post-surgery clinical complications such as risk of infection, amputation, and increased mortality. In addition, antidiabetic treatment has been associated with bone effects, in particular TZDs and SGLT2 inhibitors. Despite all the known negative consequences on bone repair and regeneration, the mechanisms involved in T2DM-induced skeletal impairment are not yet fully understood. Further investigation must be carried out to elucidate the pathophysiology of the diabetic bone and to develop successful strategies to treat this growing medical and socioeconomical global complication.

## Author Contributions

Article conception and structuration: CM, GK, and KV. Literature search, analysis, and manuscript drafting: CM. Critical revision of manuscript content: FL, BS, GK, and KV. Approving final version of manuscript: CM, FL, BS, GK, and KV. CM takes responsibility for the integrity of the data analysis.

## Conflict of Interest Statement

The authors of this study declare that the research was conducted in the absence of any commercial or financial relationships that could be construed as a potential conflict of interest.
